# Soluble Syndecan-1 Levels Are Associated with Survival in Platinum-Treated Bladder Cancer Patients

**DOI:** 10.3390/diagnostics10110864

**Published:** 2020-10-23

**Authors:** Csilla Olah, Stephan Tschirdewahn, Michèle J. Hoffmann, Ulrich Krafft, Boris Hadaschik, Peter Nyirady, Attila Szendröi, Orsolya Módos, Anita Csizmarik, Ilona Kovalszky, Henning Reis, Tibor Szarvas

**Affiliations:** 1 Department of Urology, University of Duisburg-Essen, 45147 Essen, Germany; Csilla.Olah@uk-essen.de (C.O.); Stephan.Tschirdewahn@uk-essen.de (S.T.); Ulrich.Krafft@uk-essen.de (U.K.); Boris.Hadaschik@uk-essen.de (B.H.); 2 Department of Urology, Medical Faculty, Heinrich-Heine-University Duesseldorf, 40225 Duesseldorf, Germany; Michele.Hoffmann@uni-duesseldorf.de; 3 Department of Urology, Semmelweis University, 1089 Budapest, Hungary; nyirady.peter@med.semmelweis-univ.hu (P.N.); szendroi.attila@med.semmelweis-univ.hu (A.S.); modos.orsolya@med.semmelweis-univ.hu (O.M.); csizmarik.anita@med.semmelweis-univ.hu (A.C.); 4 1st Institute of Pathology and Experimental Cancer Research, Semmelweis University, 1085 Budapest, Hungary; kovalszky.ilona@med.semmelweis-univ.hu; 5 Institute of Pathology, University of Duisburg-Essen, 45147 Essen, Germany; henning.reis@uk-essen.de

**Keywords:** muscle-invasive bladder cancer, SDC1, chemotherapy, cisplatin, metastasis, Matrix Metalloproteinase—7 (MMP-7)

## Abstract

Cisplatin-containing chemotherapy represents the first-line treatment for patients with locally advanced or metastatic muscle-invasive bladder cancer. Recently, novel therapies have become available for cisplatin-ineligible or -resistant patients. Therefore, prediction of cisplatin response is required to optimize therapy decisions. Syndecan-1 (SDC1) tissue expression and serum concentration may be associated with cisplatin resistance. Thus, pre-treatment serum levels of SDC1 and its expression in chemo-naïve tissues were assessed in 121 muscle-invasive bladder cancer patients who underwent postoperative platinum-based chemotherapy. SDC1 concentrations were evaluated by ELISA in 52 baseline and 90 follow-up serum samples and tissue expressions were analyzed by immunohistochemistry in an independent cohort of 69 formalin-fixed paraffin-embedded tumor samples. Pre-treatment SDC1 serum levels were significantly higher in lymph node metastatic (*p* = 0.009) and female patients (*p* = 0.026). SDC1 tissue expression did not correlate with clinicopathological parameters. High pre-treatment SDC1 serum level and the presence of distant metastasis were independent risk factors for overall survival (Hazard ratio (HR): 1.439, 95% Confidence interval (CI): 1.003–2.065, *p* = 0.048; HR: 2.269, 95%CI: 1.053–4.887, *p* = 0.036). Our results demonstrate an independent association between high baseline serum SDC1 concentration and poor survival in platinum-treated patients. Analyzing baseline serum SDC1 levels may help to predict platinum-containing chemotherapy and could help to optimize therapeutic decision-making.

## 1. Introduction

Urothelial bladder cancer (BC) is a common malignancy with approximately 550,000 new cases worldwide each year [1]. At first diagnosis, 70–75% of cases are non-muscle-invasive BC (NMIBC). NMIBC patients have a good prognosis with a 5-year survival rate of over 90%, a high frequency of recurrence but a rather low tendency of progression to muscle-invasive BC (MIBC). MIBC occurs in 20–25% of newly diagnosed cases and it is an aggressive malignancy with a high metastatic potential [2]. For patients with organ-confined disease, radical cystectomy (RC) alone provides a 5-year survival rate of 80%, while patients with locally advanced and/or metastatic disease, the 5-year survival rate varies widely between 15% and 50%. Thus, for locally advanced and/or metastatic MIBC patients, perioperative chemotherapy is recommended. The gold-standard treatment for eligible patients, with adequate renal function, is the cisplatin-containing combination chemotherapy; either methotrexate–vinblastine–doxorubicin–cisplatin (MVAC) or gemcitabine–cisplatin (GC), with a 40–60% response rate [3]. These two therapy regimes have similar efficacy in terms of survival, however, GC is more frequently used due to its better tolerability [4]. Recently, immune checkpoint and FGFR2/3 (Fibroblast Growth Factor Receptor 2 and 3) inhibitors have become available for platinum-resistant BC patients [5]. With available potentially effective therapies for platinum-resistant patients, molecular markers are urgently needed in order to select the type and timing of different treatments.

In the last few years, distinct gene expression-based molecular subtypes of MIBC have been established and different subtypes were suggested to show higher sensitivities to platinum-based chemotherapy [6]. However, recent studies could not confirm the same or overlapping subtypes to be more sensitive to platinum therapy [7,8]. In addition, the presence of *ERCC2* mutations was shown to be associated with a better response to platinum therapy [9]. Even though the positive predictive value of *ERCC2*-alterations is high, some patients without *ERCC2* alterations also showed favorable response to platinum therapy. In former studies, the relevance of other molecular alterations for platinum resistance was published, such as increased ERCC1 gene and protein expressions, and high survivin and HMGA2 tissue expressions were associated with poor response to platinum therapy of BC [10,11,12]. However, none of these molecular markers has been integrated into the clinical decision-making. Therefore, prediction of platinum chemotherapy is still an unmet clinical need. 

Syndecan-1 (SDC1) is a member of the transmembrane heparan sulfate proteoglycan family, which is predominantly expressed in epithelial cells. Its tissue expression, intracellular localization and the serum concentration of its shed ectodomain were shown to be associated with tumor stage, grade, and patients’ survival in BC [13,14]. Furthermore, Yu et al. demonstrated that syndecan-1 (SDC1) is significantly up-regulated and involved in platinum resistance of hepatocellular carcinoma cell lines [15]. Matrix metalloproteinase-7 (MMP-7) is one of the few molecules capable of shedding the SDC1 ectodomain region [16]. Additional data suggested the involvement of SDC1 in the resistance to systemic treatments of various malignancies [16,17,18]. 

In the case of BC, the role of SDC1 in platinum-treated patients has not been examined yet. Therefore, we determined pre-treatment serum concentrations and tissue expressions of SDC1 in BC patients who received platinum-containing chemotherapy. We also determined the serum MMP-7 level to examine the correlation with SDC1 concentration.

## 2. Materials and Methods

### 2.1. Patient Cohorts

In this study, a total of 121 patients were analyzed. Patients were divided into two cohorts. The first “ELISA cohort” included 52 BC patients, who received platinum-based chemotherapy between January/2010 and December/2017 at the Department of Urology, University Hospital of Essen (*n* = 16), and the Department of Urology of Semmelweis University of Budapest (*n* = 36). Baseline serum samples were collected postoperatively, directly before the first cycle of platinum treatment. A further 90 serum samples of 21 patients were taken during chemotherapy for serum monitoring analysis. The second “IHC (immunohistochemistry) cohort” consisted of 69 BC patients with available chemo-naïve formalin-fixed and paraffin-embedded (FFPE) tumor tissue from patients who underwent platinum-based chemotherapy between January/2004 and March/2010 at the Department of Urology, University Hospital of Essen (*n* = 33) or participated in a phase II, prospective, multicenter, randomized, double-blinded trial (*n* = 36) (SUSE, AB 31/05, RUTT 204) [19]. Inclusion criteria for study enrollment were histologically confirmed diagnosis of locally advanced and/or metastatic urothelial BC, age over 18 years, and no chemotherapy before surgery. The median time between surgery (FFPE sample collection) and chemotherapy was available for 50 of 69 patients and had a range of 68 days (IRQ: 47–95 days). All patients received transurethral resection both in the serum and the tissue cohorts. In addition, 44 of 52 patients (ELISA cohort) and 54 of 69 patients (IHC cohort) were treated with radical cystectomy before chemotherapy (curative chemotherapy groups shown in Table 1). The endpoints of the study were overall survival (OS) and progression-free survival (PFS). The endpoints were calculated as the period between first chemotherapy and survival/death or progression. The study was performed according to the ethical standards of the Helsinki Declaration and the study protocol was approved by the ethical board of the University of Duisburg-Essen and Semmelweis University of Budapest (15-6400-BO/(May 2015) and TUKEB 55/2014 (November 2013).

### 2.2. SDC1 Enzyme-Linked Immunosorbent Assay (ELISA)

Patients’ SDC1 serum levels were determined by an SDC1 ELISA kit (Diaclone CD138, Gene-Probe, San Diego, CA, USA; Cat.Nr.: 950.640.096) according to the manufacturer’s instructions. The cut-off value of SDC1 for dichotomization was set at the upper 25th percentile (180 ng/mL). Serum MMP-7 levels were measured in a previously performed study by using the Quantikine ELISA kit from R&D Systems (Wiesbaden, Germany; Cat.Nr.: DMP700) (unpublished results).

### 2.3. SDC1 Immunochemistry

A mouse monoclonal antibody against SDC1/CD138 (clone MI15, dilution 1:100, Dako/Agilent, Santa Clara, CA, USA) was used to perform IHC staining after heat-based antigen retrieval (30 min, 96°C, pH 6.0) on the 4 µm thick FFPE sections. Automated IHC was performed using the Dako Autostainer Plus System (Dako) with the anti-mouse IgG EnVision Plus detection kit (Dako) for secondary and tertiary immunoreactions. Negative controls were calculated by each run [20]. Reaction products were developed with diamino-benzidine (DAB), according to general protocols. SDC1 staining intensity was scored as 1, 2, or 3, equivalent to negative, moderate, and strong intensities. A percentage score was also defined as 0–10%—0 Pts., 11–20%—1 Pt., 21–30%—2 Pts., 31–40%—3 Pts., 41–50%—4 Pts., 51–60%—5 Pts., 61–70%—6 Pts., 71–80%—7 Pts., 81–90%—8 Pts., 91–100%—9 Pts. Finally, a score was calculated by multiplying the intensity score and percentage score. Moderate SDC1 expression was considered as a score between 4 and10, and strong expression was considered as a score higher than 12. SDC1 expression was evaluated separately for cell membrane, cytoplasm, and stroma.

### 2.4. Statistical Analysis

For paired comparisons between groups, the nonparametric, two-tailed Wilcoxon rank-sum test was applied. The associations of SDC1 immunostaining with the clinicopathological parameters were examined using a Pearson chi-squared test. Survival analyses were evaluated using a Kaplan–Meier log-rank test and univariate Cox analysis. For multivariate analysis, Cox regression models were used, including parameters with a *p*-value of <0.05 in the univariate analysis. In all tests, a *p*-value <0.05 was considered statistically significant. All statistical analyses were performed using the SPSS software package (IBM SPSS Statistics for Windows, version 25, IBM Corp., Armonk, NY, USA).

## 3. Results

### 3.1. Patients’ Characteristics

The main characteristics of patient cohorts are demonstrated in Table 1. In the ELISA cohort, patients’ median age was 65 years. Thirty-eight patients (73%) were male, and 14 (27%) were female. Forty-three patients (83%) had an ECOG (Eastern Cooperative Oncology Group) performance status (PS) of 0 and nine (17%) had a PS higher than 0. Tumor stage was available in 41 cases (79%) (1 × pT1, 9 × pT2, 21 × pT3, 10 × pT4). Thirty-four patients (65%) had lymph node metastasis alone and nine (17%) had visceral metastasis. Thirty-one patients (60%) died during the follow-up period. The median follow-up time for OS was 16.5 months, (range: 2–101 months) and for PFS, 13.4 months (range: 1–101 months). 

In the IHC cohort, the median age of patients was 64 years. Fifty-three patients (77%) were male, and 16 (23%) were female. Thirty-six patients (52%) had an ECOG performance status of 0 and 33 (48%) had a PS higher than 0. In 54 cases (78%), the tumor stage was available (1 × pT1, 15 × pT2, 26 × pT3, 12 × pT4). Twenty-nine patients (42%) had lymph node metastasis alone and 30 (44%) had visceral metastasis, while in nine cases, bone metastases were demonstrated. Forty-eight patients (70%) died during the follow-up period. The median OS was 10 months (range: 1–123 months), and the PFS was 6 months (range: 1–123 months).

### 3.2. Correlation of SDC1 Serum Levels and Tissue Protein Expressions with Clinicopathological Parameters

SDC1 serum levels were examined in pre-treatment (baseline) serum samples of 52 patients who received platinum-based chemotherapy. In addition to the 52 baseline samples, a further 90 serum samples were collected during chemotherapy. Correlations between clinicopathological parameters and baseline SDC1 concentrations are shown in Table 2. Patients’ age, tumor stage, ECOG performance status, prior cystectomy (curative vs. palliative treatment groups), and distant metastases did not correlate with the SDC1 serum concentration. However, in female patients, SDC1 serum levels were significantly increased (*p* = 0.026), moreover, SDC1 baseline values were significantly correlated with the presence of lymph node metastases as well (*p* = 0.026).

**Figure 1:**
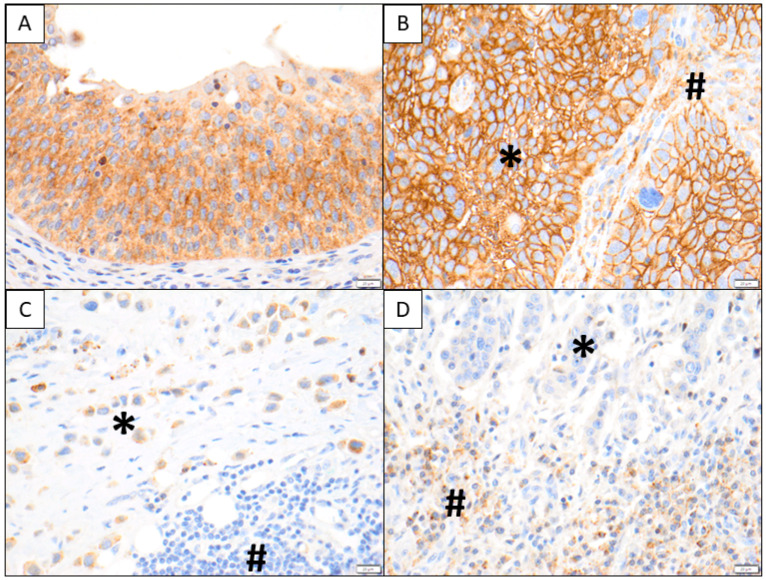
In (**A**), typical SDC1 staining is shown in non-neoplastic urothelium including intra-epithelial lymphocytes. In (**B**), strong tumoral membranous and cytoplasmic SDC1 immunoreactivity is depicted (*), while stromal elements exhibit a weak to moderate SDC1 immunoreactivity (#). In contrast, a plasmacytoid urothelial carcinoma shows weak to moderate SDC1 staining (#) in (**C**), while the stromal components and lymphocytes are SDC1 negative (#). In (**D**), only very faint SDC1 tumor cell reactivity is visible (*) and the plasma cells at the tumor–stroma interface show moderate to strong granular cytoplasmic SDC1 positivity (#).

SDC1 tissue expression was analyzed in 69 chemo-naïve tissue samples of patients who received platinum-based chemotherapy. SDC1 is known as a plasma cell marker, which was also obvious in our IHC analysis. In addition to plasma cells, membranous and cytoplasmatic SDC1 staining were characteristic for tumor cells. SDC1 staining could also be observed in some other stromal cells (Figure 1). Therefore, we examined the membranous and cytoplasmatic SDC1 intensity of tumor cells, as well as that of stromal cells separately. The correlation between the main clinicopathological parameters and SDC1 tissue expression is summarized in Table 2. SDC1 staining intensity of tumor and stromal cells did not show any significant correlation with patients’ clinicopathological parameters

### 3.3. Correlation of SDC1 Levels and Survival

**Figure 2:**
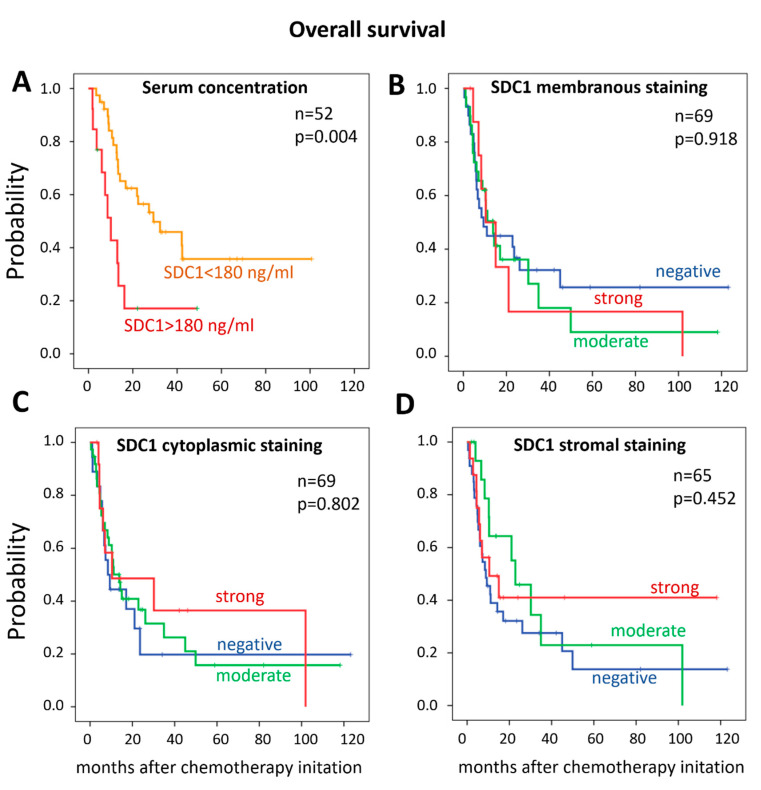
Kaplan–Meier curves of overall survival stratified by (**A**) SDC1 serum concentration (>180 ng/mL) and SDC1 tissue expression, (**B**) SDC1 membranous staining, (**C**) SDC1 cytoplasmic staining, (**D**) SDC1 stromal staining.

Univariate analysis revealed that the presence of distant metastasis was associated with shorter OS in both the ELISA and the IHC cohorts (*p* = 0.021 and *p* = 0.041, respectively). Similarly, distant metastases were significantly associated with shorter PFS (*p* < 0.001) in the ELISA cohort and tended to correlate with shorter PFS in the IHC cohort (*p* = 0.073). In addition, a higher baseline SDC1 serum concentration (>180 ng/mL) correlated with a significantly shorter OS (*p* = 0.006) (Table 3, Figure 2). Accordingly, the OS and PFS were shorter (9.0 and 8.2 months) in patients with high pre-treatment SDC1 levels, compared to those with low serum SDC1 concentrations (22 and 18.7 months, respectively). This correlation remained significant in the subgroup of patients who received cystectomy prior to chemotherapy (*p* = 0.043). SDC1 membranous, cytoplasmic, or stromal staining were not associated with OS or PFS (Table 3, Figure 2). Patients’ age, sex, tumor stage, or lymph node metastases did not have an impact on OS or PFS in any of the two cohorts. In the IHC cohort, the ECOG performance status was associated with shorter OS and PFS (*p* = 0.006 and *p* = 0.008, respectively). 

In the ELISA cohort, multivariate analysis showed that the occurrence of distant metastasis and elevated SDC1 serum levels (>180 ng/mL) are independent risk factors for OS (*p* = 0.036 and 0.048) (Table 4A). In the subgroup of patients who received cystectomy prior to chemotherapy (curative treatment group), a high SDC1 serum level also proved to be an independent predictor of shorter OS (HR: 1.353; 95%CI: 1.002–1.827; *p* = 0.049). In the IHC cohort, the multivariate analysis showed only ECOG PS as an independent risk factor for OS and PFS (Table 4B). 

Serum samples taken during the chemotherapy cycles were available for 21 patients. SDC1 concentrations were categorized as evaluated by 25% cut-off values. Nine of these 21 patients (43%) died during the follow-up period. Five of these nine patients (56%) had increasing SDC1 levels during cisplatin treatment compared to the baseline. In 10 patients (48%), progression was detected during chemotherapy or the follow-up period. Six of these 10 patients (60%) had increasing SDC1 concentrations. Eight of the 21 patients (38%) survived without progression within the follow-up period. In six of these eight patients (75%), decreasing SDC1 concentrations were detected (Supplementary Table S1) (Supplementary Figure S1).

### 3.4. Correlation Between Serum SDC1 and MMP-7 Levels

Baseline MMP-7 levels were available for 45 patients for comparison with those of SDC1. According to Spearman’s rho test, higher serum SDC1 level significantly correlated with higher MMP-7 level (correlation coefficient: 0.326, *p* = 0.029) (Supplementary Figure S2). Moreover, high serum concentration of MMP-7 (>10 ng/mL) proved to be an independent risk factor for OS (HR: 4.547, CI: 1.067–4.702, *p* = 0.033) when adjusted for distant metastasis.

## 4. Discussion

In the present study, we retrospectively examined serum concentrations and tissue expressions of SDC1 in chemo-naïve samples of BC patients who underwent platinum-containing chemotherapy. Our results demonstrate that high pre-treatment serum SDC1 levels are independently associated with poor survival in platinum-treated BC patients. 

Patients with locally advanced and/or metastatic BC have a heterogeneous response to the gold-standard cisplatin-based chemotherapy. Recently, immunotherapies and targeted therapies, such as FGFR inhibitors have become available for cisplatin-resistant patients. Therefore, biomarkers are required to predict patients’ individual responses to platinum therapy, in order to optimize therapeutic decision-making. 

SDC1 is involved in numerous cellular processes by its ectodomain heparan sulfate chain, which is able to bind numerous regulatory molecules such as cytokines, growth factors, and extracellular components. By interacting with these molecules, it plays a role in downstream signaling pathways, affecting morphogenesis, cell migration, invasion, EMT (epithelial-to-mesenchymal transition), and angiogenesis [16,21].

SDC1 is expressed by normal epithelial and endothelial cells and by carcinoma cells as well. Altered SDC1 expression has been associated with the presence and progression of various tumors [22,23], including BC [13,14]. Progression of epithelial tumors is frequently accompanied by the loss of SDC1 expression as part of the epithelial-to-mesenchymal transition [24]. Furthermore, increased SDC1 expression of stromal cells was reported as a risk factor for poor clinical outcomes in different tumor types [25]. Miyake et al. showed that high-grade BC tumors exhibit increased cytoplasmic SDC1 expression and a parallel decrease in its membranous expression [13]. In line with this observation, we previously found decreased membrane SDC1 expression in BC cells and a simultaneous elevated stromal SDC1 staining in high-stage and poorly differentiated BC. Accordingly, a high level of SDC1 stromal staining was independently associated with poor OS [14]. Previously, independent studies in various tumor types examined the potential influences of SDC1 on the efficacy of different chemotherapeutic agents. Yu et al. examined the impact of SDC1 on cisplatin resistance in hepatic cancer and demonstrated that SDC1 gene and protein expression were up-regulated in cisplatin-resistant cell lines. Moreover, they measured an increased cisplatin sensitivity in SDC1-depleted cells. They found that SDC1 stimulates the PI3K/AKT signaling pathway, which is known to be involved in cisplatin resistance [15]. In accordance with previously published results in chemo-naïve patients, the correlation between high stromal SDC1 expression and higher tumor-specific mortality was also described in patients with oral squamous carcinoma who received neoadjuvant cisplatin-containing chemotherapy [26]. In the present study, we determined SDC1 expression in different cellular (tumoral vs. stromal) and subcellular (membranous vs. cytoplasmic) localizations, but found no significant correlations between SDC1 tissue expressions and PFS or OS in our platinum-treated BC patients. This observation is different to that of what we previously found in BC of various stages [14]. One possible explanation for this difference is that our present study included a highly selective patient cohort with advanced, chemotherapy-treated BC patients, while our previous study included BC patients of all tumor stages (including pTa and pT1 tumors as well) [14]. Based on these observations, the shedding of SDC1 from the tumor cell surface, and consequently its appearance in the tumor stroma, may be associated with tumor aggressiveness, invasion, and progression but, in the meantime, it does not seem to be predictive for platinum-based chemotherapy. Functional studies may provide a deeper insight into the possible role of serum and tissue SDC1 for therapy response. Recently, Seiler et al. demonstrated that the glycosaminoglycan chains of SDC1 may also have an impact on platinum sensitivity of BC patients, highlighting that not only the primary protein expression but also the glycosaminoglycan component of SDC1 may be important [27].

The extracellular domain of SDC1, bearing the core protein and heparan sulfate chains, can be proteolytically removed from the cell surface. As a consequence of this ectodomain shedding, SDC1 can be measured in serum samples. Increased SDC1 serum levels were demonstrated in various tumors, such as myeloma, breast cancer, and colon carcinoma [21]. In BC, we previously demonstrated that high preoperative serum levels of SDC1 are associated with progressed tumor stages, the presence of lymph node metastases, and are independently associated with poor patient survival [14]. Anttonen et al. described a significant association between elevated pre-treatment serum SDC1 and poor prognosis in small cell lung cancer patients treated with platinum-containing therapy [18]. Further studies reported that SDC1 shedding is enhanced as a response to chemotherapy and shed SDC1 was found to be associated with chemotherapy resistance in colorectal cancer [16]. Similarly, we demonstrated high circulating pre-treatment SDC1 levels to be independently associated with poor response to docetaxel chemotherapy in patients with castration-resistant prostate cancer [17]. Our present analyses revealed for the first time an independent association between high baseline serum SDC1 concentrations and shorter OS in platinum-treated BC patients. Therefore, soluble SDC1 levels may help to identify platinum-treated patients who are at high risk of early death. Further research should clarify whether these patients may benefit from other available and potentially effective therapies.

Ramani et al. reported that chemotherapeutic drugs can induce the expression and shedding of SDC1 in multiple myeloma and pancreatic cancer cells [28]. The soluble SDC1 ectodomain remains a biologically active molecule, performs its functions, and binds the same ligands, and therefore shed SDC1 continues to be involved in signal transduction processes [21]. The heparan sulfate chains of soluble SDC1 are able to bind heparin-binding epithelial growth factor, thereby activating the EGFR (Epidermal Growth Factor Receptor) downstream signaling pathway and may contribute to resistance development against chemotherapy [16]. Therefore, chemotherapy-induced changes in soluble SDC1 levels might be informative for treatment effectivity and prognosis. Thus, we assessed SDC1 changes during platinum therapy and correlations with disease progression and patient survival. Although, in some cases, SDC1 concentrations showed remarkable changes during platinum therapy, the small number of cases (*n* = 21) did not allow for valid evaluation of the association between SDC1 changes and PFS or OS. 

Studies in multiple myeloma and breast and colon cancer detected specific proteases, including MMP-7, which is synthetized by the tumor cells, and is able to proteolytically remove the ectodomain of SDC1 [21]. In accordance, we previously found in two independent patient cohorts of bladder and prostate cancer that high soluble serum SDC1 levels are directly associated with elevated MMP-7 serum levels [14,17], which suggests MMP-7 as a major protease for SDC1 shedding. In the present study, we also detected a positive correlation between serum SDC1 and previously measured MMP-7 levels in BC, which further confirmed the role of MMP-7 in SDC1 shedding.

## 5. Conclusions

Our present data revealed the independent prognostic value of serum SDC1 in patients with advanced BC who underwent chemotherapy. Our results provide a basis for further analysis regarding the potential involvement of SDC1 in platinum resistance of bladder cancer. In addition, further research is needed to test the predictive value of SDC1 in platinum-treated BC patients.

## Figures and Tables

**Table 1:** Patients’ characteristics. ECOG—Eastern Cooperative Oncology Group, PS—performance status. Gem/Cis—gemcitabine + cisplatin, Gem/Carbo—gemcitabine + carboplatin, MVAC—methotrexate + vinblastine plus + doxorubicin + cisplatin.

Variables	ELISA Cohort	IHC Cohort
	*n* (%)	*n* (%)
Total number of patients	52	69
Age at baseline, median (range)	65 (41–81)	64 (37–90)
≤65	27 (52)	33 (48)
>65	25 (48)	36 (52)
Sex		
Male	38 (73)	53 (77)
Female	14 (27)	16 (23)
ECOG PS at enrollment		
0	43 (83)	36 (52)
1	8 (15)	28 (41)
2–3	1 (2)	5 (7)
Stage		
pT1	1 (2)	1 (1)
pT2	9 (17)	15 (22)
pT3	21 (40)	26 (38)
pT4	10 (20)	12 (17)
n.a.	11 (21)	15 (22)
Metastases		
Lymph node metastasis (>2 cm)	34 (65)	29 (42)
Distant metastasis	12 (23)	39 (57)
Soft tissue lesions (lung/liver)	9 (17)	30 (44)
Bone metastasis	3 (6)	9 (13)
Chemotherapy regimen		
Gem/Cis	47 (90)	57 (83)
Gem/Carbo	5 (10)	-
MVAC	-	12 (17)
Curative (prior cystectomy)	44 (85)	54 (78)
Palliative (no prior cystectomy)	8 (15)	15 (22)
Number of cycles, median [range]	3 [1,2,3,4,5,6,7,8,9]	4 [1,2,3,4,5,6,7,8]
Single (only one series)	4	10
Collection site		
Essen	16 (31)	33 (48)
Budapest	36 (69)	-
SUSE	-	36 (52)
Number of patients died	31 (60)	48 (70)
Follow-up time in months, median (range)	17 (2–101)	10 (1–123)

**Table 2:** Syndecan-1 (SDC1) serum and tissue levels and clinicopathological parameters. ECOG—Eastern Cooperative Oncology Group. Bold printed *p*-values were significant (<0.05).

Variables	ELISA cohort			IHC Cohort																	
		Serum SDC1 Concentration			SDC1 Expression (staining score)						SDC1 Expression (staining score)							SDC1 Expression (staining score)			
					**membrane**						**cytoplasm**							**stroma**			
	*n*	median (range)	*p*	*n*	0	%	>0	%	*p*	*n*	0	%	>0	%	*p*	*n*	0	%	>0	%	*p*
Age 65 (41–81)																					
≤65	27	72.3 (8.3–1099.9)	0.810	37	17	46	20	54	0.657	37	12	32	25	68	0.328	36	19	53	17	47	0.718
>65	25	73.3 (23.4–1120.8)		32	13	41	19	59		32	7	22	25	78		29	14	48	15	52	
Sex																					
Male	38	72.2 (8.4–356.4)	**0.026**	53	24	45	29	55	0.582	53	15	28	38	72	0.769	49	26	53	23	47	0.518
Female	14	91.8 (8.3–1120.8)		16	6	38	10	62		16	4	25	12	75		16	7	44	9	56	
Stage																					
pT1–pT2	10	60.5 (8.4–356.4)	0.577	16	6	38	10	62	0.400	16	5	33	11	67	0.866	16	8	50	8	50	0.846
pT3–pT4	31	72.4 (19.3–1120.8)		38	19	50	19	50		38	11	29	27	71		34	18	53	16	47	
Not available	9			15						15						15					
ECOG status																					
0	43	72.4 (8.3–1120.8)	0.227	36	12	33	24	67	0.076	36	7	19	29	81	0.116	35	20	57	15	43	0.267
≥1	9	67.8 (19.3–305.3)		33	18	55	15	45		33	12	36	21	64		30	13	43	17	57	
Lymph node status																					
N0	10	182.5 (23.4–1120.8)	**0.026**	25	11	46	14	56	0.753	25	10	40	15	60	0.121	24	13	54	11	46	0.768
N+	34	61.9 (8.3–356.4)		29	14	48	15	52		29	6	21	23	79		25	13	52	13	48	
Nx	8			15						15						15					
Distant metastasis																					
Absent	40	72.2 (8.4–356.4)	0.312	35	18	51	17	49	0.305	35	9	26	26	74	0.392	32	17	53	15	47	0.832
Present	12	91.8 (8.3–1120.8)		19	7	37	12	63		19	7	37	12	63		18	9	50	9	50	
Not available				15						15						15					
Collection site																					
Budapest	36	75.8 (8.3–1120.8)	0.677	-						-						-					
Essen	16	62.4 (19.7–373.2)		36	17	47	19	53	0.512	36	9	25	27	75	0.622	33	15	45	18	55	0.384
SUSE	-			33	13	39	20	61		33	10	30	23	70		32	18	56	14	44	

**Table 3:** Cox univariate overall survival (OS) and progression-free survival (PFS) analysis in the ELISA (serum SDC1 concentration) and IHC cohort (SDC1 expression). HR—hazard ratio, CI—confidence interval, ECOG — Eastern Cooperative Oncology Group. Bold printed *p*-values were significant (<0.05).

Cox Univariate Analysis												
		ELISA Cohort					IHC Cohort					
Variables	Overall Survival			Progression-Free Survival			Overall Survival			Progression-Free Survival		
	HR	95% CI	*p*	HR	95% CI	*p*	HR	95% CI	*p*	HR	95% CI	*p*
Age >65	1.119	0.553–2.266	0.755	0.403	0.142–1.145	0.088	1.292	0.722–2.312	0.388	1.358	0.790–2.344	0.268
Female	0.648	0.266–1.581	0.340	0.934	0.303–2.877	0.906	1.460	0.718–2.969	0.296	1.396	0.732–2.664	0.311
Invasive stage (≥pT2)	0.659	0.279–1.558	0.342	0.760	0.234–2.474	0.649	1.049	0.506–2.175	0.897	1.292	0.630–2.649	0.484
ECOG status (≥1)	1.391	0.703–2.757	0.343	1.641	0.804–3.349	0.173	2.306	1.276–4.169	**0.006**	2.088	1.209–3.606	**0.008**
Lymph node positivity	0.990	0.369–2.655	0.983	1.838	0.414–8.158	0.423	0.542	0.285–1.030	0.061	0.598	0.322–1.108	0.102
Distant metastasis	2.459	1.147–5.273	**0.021**	7.832	2.627–23.349	**<0.001**	1.881	1.026–3.446	**0.041**	1.662	0.954–2.894	0.073
Serum SDC1 >180 ng/mL	1.433	1.109–1.852	**0.006**	1.236	0.868–1.758	0.240						
Membrane SDC1 positivity							1.165	0.652–2.080	0.606	0.917	0.538–1.564	0.750
Cytoplasm SDC1 positivity							0.777	0.415–1.453	0.429	0.951	0.517–1.751	0.873
Stroma SDC1 positivity							0.685	0.377–1.245	0.214	0.837	0.485–1.446	0.524

**Table 4:** Cox multivariate OS and PFS analysis in the ELISA (**A**—serum SDC1 concentration) and IHC cohort (**B**—SDC1 expression). HR—hazard ratio, CI—confidence interval, ECOG—Eastern Cooperative Oncology Group. Bold printed *p*-values were significant (<0.05).

Cox multivariate Analysis						
A	ELISA Cohort					
**Variables**	**Overall survival**			**Progression-free survival**		
	HR	95% CI	*p*	HR	95% CI	*p*
Distant metastasis	2.269	1.053–4.887	**0.036**	8.107	2.590–25.374	**<0.001**
Serum SDC1 >180 ng/mL	1.439	1.003–2.065	**0.048**	0.945	0.557–1.604	0.834
**B**	**IHC cohort**					
**Variables**	**Overall survival**			**Progression-free survival**		
	HR	95% CI	*p*	HR	95% CI	*p*
Distant metastasis	1.373	0.715–2.636	0.340	1.294	0.691–2.423	0.421
ECOG status (≥1)	3.019	1.524–5.982	**0.002**	2.158	1.14–4.086	**0.018**

## Supplementary Materials

Supplementary materials can be found at https://www.mdpi.com/2075-4418/10/11/864/s1. Table S1. Changes of SDC1 serum levels during platinum-containing chemotherapy. Figure S1. Changes of SDC1 serum levels during platinum-containing chemotherapy. Figure S2. Correlation between SDC1 and MMP-7 serum concentrations.

## Abbreviations

BC	bladder cancer
CI	confidence interval
ECOG	Eastern Cooperative Oncology Group
FFPE	formalin-fixed and paraffin-embedded
GC	gemcitabine + cisplatin
HR	hazard ratio
IHC	immunohistochemistry
MIBC	muscle-invasive bladder cancer
MMP-7	matrix metalloproteinase-7
NMIBC	non-muscle-invasive bladder cancer
OS	overall survival
PFS	progression-free survival
SDC1	syndecan-1
MVAC	methotrexate + vinblastine plus + doxorubicine + cisplatin
