# Supplementary material for: Soluble Syndecan-1 Levels Are Associated with Survival in Platinum-Treated Bladder Cancer Patients
Source: Diagnostics (Basel). 2020 Oct 23;10(11):864. doi: 10.3390/diagnostics10110864 (PMC7690724; doi:10.3390/diagnostics10110864)
Supplement: Supplementary file 1 [file diagnostics-10-00864-s001.pdf]

diagnostics

MDPI

Table S1. Changes of SDC1 serum levels during platinum-containing chemotherapy. OS: overall survival, PFS: progression-free survival, Prog.: progression, C: number of chemotherapy cycles, D: day of the chemotherapy cycle.

	OS		PFS		SDC1 Serum Levels During Platinum Therapy (C—cycle / D—day)											
**Sample Number**	**Death**	**Months**	**Prog.**	**Months**	**C1/D1**	**C1/D8**	**C2/D1**	**C2/D8**	**C3/D1**	**C3/D8**	**C4/D1**	**C4/D8**	C5/D1	C5/D8	C6/D1	C6/D8
1.	1	2	1	1	268	314	-	-	-	-	-	-	-	-	-	-
2.	0	7	1	3	77	156	87	84	90	76	-	-	-	-	-	-
3.	1	14	1	4	19	19	28	23	24	20	-	-	-	-	-	-
4.	0	42	1	5	8	8	8	55	-	63	111	116	-	-	-	-
5.	1	9	1	7	110	46	32	25	30	24	-	-	42	35	147	204
6.	1	9	1	8	67	-	155	-	109	107	-	-	-	-	-	-
7.	1	9	1	8	305	25	51	33	360	356	260	280	277	270	-	-
8.	1	27	1	22	61	-	-	40	15	-	-	-	-	-	-	-
9.	0	43	1	26	54	85	44	68	221	178	159	128	-	-	-	-
10.	0	43	1	39	21	-	14	17	-	-	-	-	-	-	-	-
11.	0	4	0	4	188	50	-	-	-	-	-	-	-	-	-	-
12.	1	6	0	6	305	58	-	-	-	-	-	-	-	-	-	-
13.	0	6	0	6	75	106	-	-	-	-	-	-	-	-	-	-
14.	1	13	0	13	92	88	112	256	-	-	-	-	-	-	-	-
15.	0	18	0	18	109	94	50	8	8	8	-	-	-	-	-	-
16.	1	22	0	22	61	97	41	-	-	-	-	-	-	-	-	-
17.	0	28	0	28	125	130	95	65	-	-	-	-	-	-	-	-
18.	0	28	0	28	55	-	68	47	-	-	-	-	-	-	-	-
19.	0	33	0	33	58	73	84	76	108	-	-	-	-	-	-	-
20.	0	33	0	33	60	31	48	59	26	25	-	-	-	-	-	-
21.	0	49	0	49	195	127	102	95	158	237	-	-	-	-	-	-

no changes compared to the baseline value (cut-off 25%)
Decreased SDC1 level after cisplatin therapy
Increased SDC1 level after cisplatin therapy
Baseline SDC1 >180 ng/mL

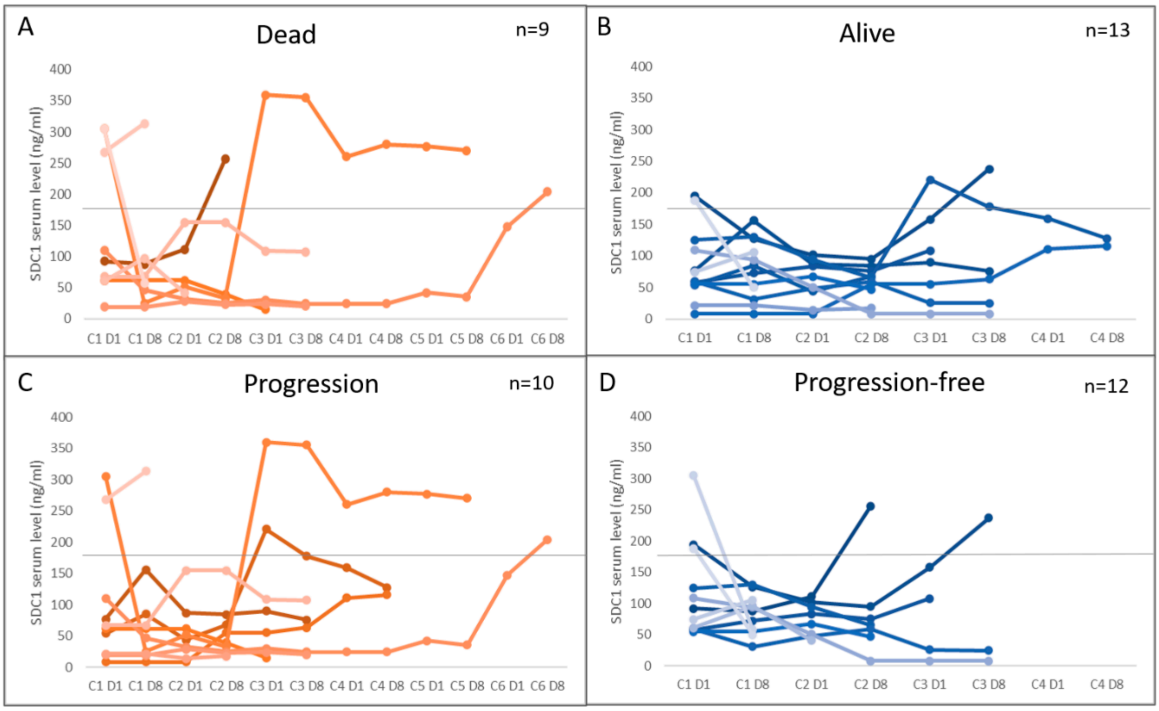

Figure S1. Changes of SDC1 serum levels during platinum-containing chemotherapy. Red curves represent patients who have died (**A**) or progressed (**C**) during the follow-up period. Blue curves represent SDC1 levels of patients alive (**B**) and without progression (**D**) at last follow-up. Grey horizontal lines represent the 180 ng/mL SDC1 concentration, the cut-off value of high SDC1 serum level before chemotherapy. (**C**): cycle, (**D**): day.

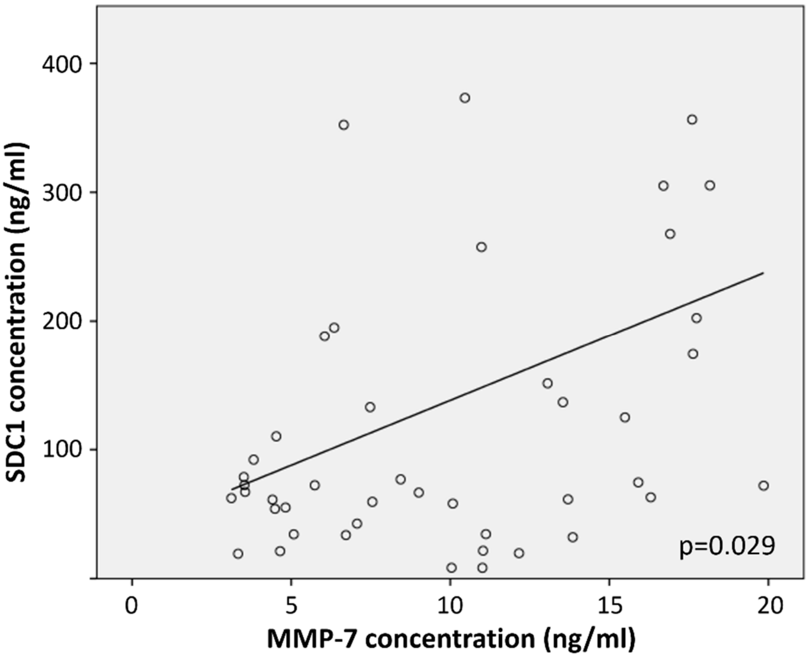

Figure S2. Correlation between SDC1 and MMP-7 serum concentrations.
